# A novel resource for studying function and dysfunction of α-synuclein: mouse lines for modulation of endogenous Snca gene expression

**DOI:** 10.1038/srep16615

**Published:** 2015-11-13

**Authors:** Natalia Ninkina, Natalie Connor-Robson, Alexey A. Ustyugov, Tatiana V. Tarasova, Tatyana A. Shelkovnikova, Vladimir L. Buchman

**Affiliations:** 1 School of Biosciences, Cardiff University, Museum Avenue, Cardiff, CF10 3AX, United Kingdom; 2 Institute of Physiologically Active Compounds Russian Academy of Sciences, 1 Severniy proezd, Chernogolovka 142432, Moscow Region, Russian Federation

## Abstract

Pathological modification of α-synuclein is believed to be an important event in pathogenesis of Parkinson’s disease and several other neurodegenerative diseases. In normal cells this protein has been linked to many intracellular processes and pathways. However, neither normal function of α-synuclein in neuronal and certain other types of cells nor its exact role in the disease pathogenesis is well understood, which is largely due to limitations of animal models used for studying this protein. We produced and validated several novel mouse lines for manipulating expression of the endogenous *Snca* gene coding for α-synuclein. These include a line for conditional Cre-recombinase-driven inactivation of the gene; a line for conditional Flp-driven restoration of a neo-cassete-blocked α-synuclein expression; a new line with a “clean” constituent knockout of the gene as well as a line carrying this knockout locus and *Rosa26-stop-lacZ* reporter locus linked at the same mouse chromosome 6. Altogether these lines represent a set of new useful tools for studies of α-synuclein normal function and the role of this protein in disease pathogenesis.

Pathological aggregation of α-synuclein with formation of characteristic intracellular inclusions is a common feature of Parkinson’s disease (PD) and several neurodegenerative diseases, collectively known as synucleinopathies^1^,^2^,^3^. Moreover, certain structural modifications or increased expression of this protein have been associated with the development of early onset forms of PD^4^,^5^,^6^,^7^,^8^,^9^, whereas polymorphisms in the encoding locus, *SNCA*, have been found to affect the risk of the development of PD and certain other synucleinopathies^10^,^11^,^12^,^13^,^14^. Therefore, it is feasible that substantial changes in α-synuclein metabolism lead to the development of aggressive forms of the disease, while more subtle modifications can be efficiently compensated for a long time. Only later in life, probably in combination with certain environmental factors and/or with a general functional decline of the ageing nervous system, these subtle modifications trigger pathological changes and clinical manifestation of the disease.

A well-documented mechanism of α-synuclein-induced neuronal dysfunction and death is the toxicity of intermediate products of α-synuclein aggregation, namely oligomers and protofibrils (reviewed in^15^,^16^,^17^,^18^). However, this gain-of-function mechanism might be accompanied by a loss-of-function developing as the result of α-synuclein sequestering in aggregates and consequent depletion of the functional pool of this protein.

α-synuclein is abundant in presynaptic terminals of many types of neurons where it might play an important role in neurotransmission via regulation of synthesis, release and re-uptake of various neurotransmitters^19^,^20^,^21^,^22^,^23^,^24^,^25^,^26^,^27^,^28^,^29^,^30^. Although α-synuclein deficiency in mouse models can be compensated for long time either due to the redundancy within the synuclein family or by switching on some other mechanism in the developing nervous system, ageing animals lacking α-synuclein develop neuronal and particularly, synaptic deficiency^31^,^32^,^33^,^34^. Depletion of α-synuclein in adult rat substantia nigra by injection of siRNA-encoding AAV viruses caused a rapid development of substantially more severe neuronal dysfunction than observed in any of the previously produced animal lines with constituent inactivation of the *Snca* gene^35^. Another limitation of conventional α-synuclein knockout mouse models is that inactivation of the gene takes place in all body cells. Despite a particular abundance of α-synuclein in neurons, it is not possible to exclude a systemic effect of its depletion in other cells, e.g. erythrocytes, on animal physiology, which might hamper interpretation of the observed phenotypes. Therefore, the ability to targetedly and conditionally deplete specific cell population of α-synuclein is important for better understanding of this protein normal function and its role in human pathologies.

Having this in mind, we produce mice for conditional Cre-recombinase-driven inactivation of the *Snca* gene. In the process of producing and characterisation of these mice we created additional mouse lines, namely mice carrying inactivated *Snca* gene which expression can be conditionally restored by FLP-recombinase-driven deletion of the neo-cassette; “clean KO” mice, a novel line with constituent inactivation of *Snca* gene that carry neither neo-cassette nor other ectopic sequences that might interfere with the genome function; mice with the same “clean KO” linked with *Rosa26-stop-lacZ* reporter locus, which is located at the same mouse Chr 6.

## Results and Discussion

**Figure 1:**
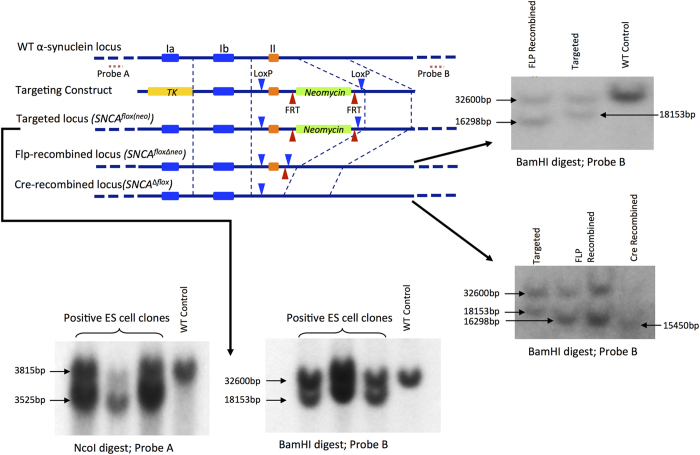
Gene targeting strategy for production of mice for conditional inactivation of α-synuclein gene and confirmation of introduced genomic modifications by Southern hybridisation. See Supplementary Fig. S1 for sequences of the wildtype (WT) and modified Snca loci.

**Figure 2:**
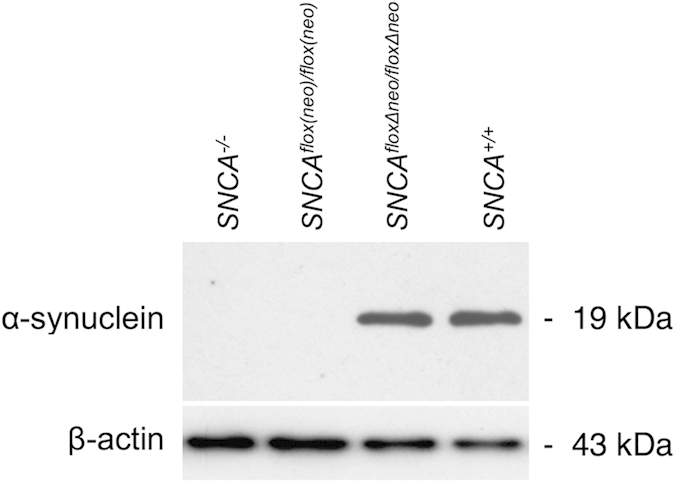
Restoration of α-synuclein expression in the cerebral cortex of floxed mice following germline deletion of neo-cassette. Western blot analysis of total protein samples extracted from the cerebral cortex of wild type mice *(SNCA*^+*/*+^), α-synuclein knockout mice *(SNCA*^−/−^, described previously^19^), floxed mice before deletion of neo-cassette *(SNCA*^*flox(neo)/flox(neo)*^) and floxed mice after deletion of neo-cassette *(SNCA*^*floxΔneo/floxΔneo*^).

**Figure 3:**
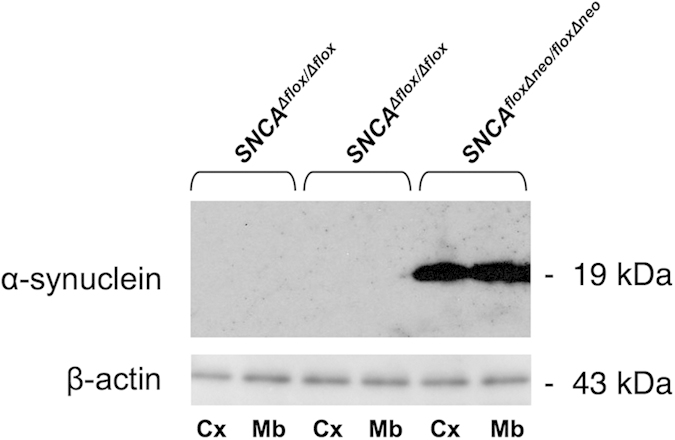
Depletion of α-synuclein in the brain tissues of homozygous mice following germline Cre-recombination. Western blot analysis of total protein samples extracted from the cerebral cortex (Cx) and midbrain (Mb) of a homozygous *(SNCA*^*floxΔneo/floxΔneo*^) α-synuclein floxed mouse after deletion of the neo-cassette and two homozygous α-synuclein floxed mice after deletion of the exon II by Cre/loxP recombination *(SNCA*^*Δflox/Δflox*^).

Using traditional gene targeting approaches schematically illustrated in Fig. 1, we produced a novel line of mice than can be used for conditional inactivation of α-synuclein coding gene by Cre-driven recombination within transcriptionally active *Snca*^*floxΔneo*^ locus. The normal level of α-synuclein in the neural tissues of homozygous *Snca*^*floxΔneo/floxΔneo*^ mice was confirmed by Western blot analysis (Fig. 2). To prove that deletion of the exon II by Cre recombination leads to complete inactivation of the gene we bred *Snca*^*floxΔneo/floxΔneo*^ mice with an “early deletor” Cre mice (see Material and Methods) to achieve germline deletion of the floxed exon II and surrounding sequences. Homozygous *Snca*^*Δflox/Δflox*^ mice were produced and Western blot analysis did not reveal any α-synuclein protein in neural tissues of these animals (Fig. 3). Therefore, successful Cre recombination within the engineered *Snca*^*floxΔneo/floxΔneo*^ locus efficiently inactivates α-synuclein expression. The line of mice carrying *Snca*^*floxΔneo*^ allele has been deposited to The Jackson Laboratory (C57BL/6-Snca<tm1.1 Vlb>/J; JAX Stock#025636). Breeding of these animals with various Cre transgenic mice or stereotaxic injection of Cre-expressing viral vectors shall make it possible to deplete specific cell population or brain areas of α-synuclein. By using tamoxifen-inducible Cre-ERT2 transgenes this inactivation process can be initiated at any period of the animal development.

However, conditional inactivation of a gene in a selected cell population commonly requires a reporter for monitoring efficiency of Cre recombination. This is particularly important for α-synuclein that in most neurons is localised not in the perikarion of targeted neurons but in presynaptic terminals, which makes it difficult to confirm its selective and efficient depletion. *Rosa26-stop-lacZ* reporter^36^ is widely used for this purpose. However, in the mouse genome both *Snca* and *Rosa26* loci are located at chromosome 6 (Chr6) approximately 50 Mb apart. To make possible the use of *Rosa26-stop-lacZ* reporter in experiments with conditional inactivation of *Snca* gene we produced a mouse line carrying a completely inactivated *Snca*^*Δflox*^ gene and *Rosa26-stop-lacZ* reporter linked at the same mouse Chr6 (see Materials and Methods). When control for the efficiency of Cre recombination is required, these mice and *Snca*^*floxΔneo*^ mice could be used for production of an experimental cohort carrying an active floxed copy of *Snca* gene at one Chr6, and a constitutively inactive copy and *Rosa26-stop-lacZ* reporter on another Chr6. In addition to the possibility of monitoring the process of Cre recombination, the efficiency of the gene inactivation process is increased when such heterozygous animals are used, because to achieve a complete knockout in a given cell, only one allele needs to be inactivated. It should be noted that there is no evidence for any noticeable effects of *Snca* gene haploinsufficiency in mice.

The line of mice with constituent inactivation of *Snca* gene (*Snca*^*Δflox/Δflox*^) has a value of its own because for the best of our knowledge, it represents the most “clean” full α-synuclein knockout produced so far. Other published mouse lines still carry various selection cassettes in the vicinity of the inactivated *Snca* gene^19^,^20^,^37^,^38^ and the natural mutant line from Harlan UK^39^ possesses a deletion of approximately 350 kb fragment that includes *Snca* along with other genes. In contrast, the only ectopic sequence remained in the new *Snca*^*Δflox/Δflox*^ line is a single loxP site and the deletion is restricted to the 1164 bp fragment containing exon II and adjacent intronic sequences of the *Snca* gene. The importance of using animals with less invasive genetic manipulations for accurate revealing the effects of α-synuclein depletion can be illustrated by the fact that in a widely used mouse knockout line^19^ the level of neuronal expression of the *Mmrn1* gene (encoding multimerin 1) located close to the modified *Snca* gene is increased when compared to wild type or our *Snca*^*Δflox/Δflox*^ mice (our unpublished observations), while in the Harlan UK mutant line this gene is completely absent. In both cases potential effects of these expression changes on neuronal physiology can be erroneously attributed to the absence of α-synuclein.

Analysis of α-synuclein expression in the nervous system of homozygous *Snca*^*flox(neo)/flox(neo)*^ mice generated at the first step of the targeting programme (see Fig. 1) revealed that the presence of the neo-cassette in the gene intron completely abolished α-synuclein production (Fig. 2). However, consequent Flp-driven deletion of this cassette restored normal expression of the protein in *Snca*^*floxΔneo/floxΔneo*^ mice (Fig. 2). Therefore, *Snca*^*flox(neo)/flox(neo)*^ line can be used for conditional switching on endogenous α-synuclein expression in specific cell populations and/or developmental periods. This might be achieved by breeding mice of this line with transgenic mice expressing Flp recombinase under control of specitic promoters or by using stereotaxic injections of Flp expressing viral vectors.

In summary, we have produced a set of unique mouse lines for conditional switching off or switching on expression of endogenous α-synuclein as well as a novel improved line with constituent inactivation of the α-synuclein encoding gene. These lines represent new useful tools for studies of the normal function of α-synuclein in selected populations of neuronal and non-neuronal cells as well as the role of this protein in the development of neurodegenerative processes and transmission of α-synuclein pathology through the nervous system.

## Materials and Methods

### Targeting construct

To create arms for homologous recombination (for sequences see Supplementary Materials and Methods), fragments from the region of the exon II of mouse *Snca* gene (encoding amino acids 1–41 of α-synuclein) were PCR amplified using DNA extracted from C57Bl6-derived ES cells (line JM8A3.N1, gift of Dr. William Skarnes, Sanger Institute, Cambridge, UK) as a template. A high fidelity AccuPrime *Pfx* SuperMix (Invitrogen) and primers carrying desired additional sequences (loxP, FRT and/or restriction endonuclease cleavage sites) were used. PCR fragments were then cloned into the pCR-Blunt II-TOPO vector (Invitrogen) and verified by sequencing. Following digestion with appropriate restriction endonucleases, resulting engineered DNA fragments were cloned into pPNT1 vector plasmid to produce final targeting construct schematically shown in Fig. 1.

### Gene targeting in mouse ES cells and generation of mouse founders

Mouse ES JM8A3.N1 cells^40^ were electroporated with the NotI-linearised targeting plasmid followed by selection of G418 and gancyclovir resistant clones. DNA extracted from individual clones was analysed for homologous recombination by Southern hybridisation with two outside probes (for sequences see Supplementary Fig. S1) as shown in Fig. 1. DNA extraction, Southern blotting, ^32^P-labeling of probes, hybridisation and washes were carried out according to protocols described previously^41^. Positive clones were expanded and used for injection into blastocysts of C57Bl6 cells followed by the embryotransfer into the uterus of pseudopregnant CD1 mice. Resulting chimeras were bred with C57Bl6 mice and successful germline transfer of the modified locus was confirmed by PCR analysis of DNA from ear biopsies.

### Production of experimental mouse lines

Mice carrying a targeted α-synuclein locus (*Snca*^*flox(neo)/*+^) were backcrossed with C57BL/6 mice for five generations and then intercrossed to produce animals homozygous for this locus (*Snca*^*flox(neo)/ flox(neo)*^) and wild type littermates (*Snca*^+*/*+^).

To remove the neo-cassette, mice carrying a targeted α-synuclein locus were bred to C57BL/6 mice for two generations and then crossed to transgenic mice expressing FLP recombinase under control of beta-actin promoter (B6.Cg-Tg(ACTFLPe)9205Dym/H, on the C57BL/6 genetic background, The Jackson Laboratory). Successful deletion of FRT-flanked neo-cassette was confirmed by Southern hybridisation and PCR analysis of DNA from ear biopsies.

Mice with deleted neo-cassette (*Snca*^*floxΔneo/*+^) were further bred to C57BL/6 J mice and selection was applied to remove the FLP recombinase transgene. Homozygous (*Snca*^*floxΔneo/floxΔneo*^) and wild type littermates were produced by the intercrossing of mice that went through seven generations of the backcrossing.

To produce mice with constituent inactivation of α-synuclein gene *Snca*^*floxΔneo/floxΔneo*^ mice were crossed with transgenic mice (on C57Bl6 background) expressing Cre recombinase under control of CMV promoter^42^. Mice with deleted neo-cassette (*Snca*^*Δflox/*+^) were further backcrossed to C57BL/6J mice and those lacking the Cre recombinase transgene were selected. Intercrosses of these mice produced homozygous (*Snca*^*Δflox/Δflox*^) animals.

Mice carrying *Snca*^*Δflox*^ and *Rosa26-stop-lacZ* reporter^36^ loci linked at the same chromosome as the result of natural crossover were selected from a pool of animals generated during crossbreeding of two mouse lines (both on C57Bl6 background), each bearing one of these modified loci.

### Animal genotyping

Animal genotypes were verified by PCR analysis of DNA from ear biopsies using combinations of primers and amplification conditions described in Supplementary Materials and Methods.

### Western blotting

Total protein samples were prepared by homogenisation of mouse tissues in the SDS-PAGE loading buffer following incubation for 10 min at 100 ^o^C. Gel electrophoresis, transfer to PVDF membrane, incubation with primary and secondary HRT-conjugated antibodies, and chemiluminescent-based detection were performed as described previously^32^. For detection of α-synuclein mouse monoclonal antibody (BD 610786 from BD Biosciences) were used in 1:1000 dilution. To confirm the equal loading membranes were re-probed with mouse monoclonal anti-beta-actin antibody (AC-15 from Sigma-Aldrich) in 1:10000 dilution.

All animal work was carried out in accordance with the United Kingdom (Scientific Procedures) Act (1986) and European Directive EC 86/609, and has been approved by the Cardiff University Ethical Review Committee and the Home Office (Project Licence 30/2844).

## Additional Information

**How to cite this article**: Ninkina, N. *et al.* A novel resource for studying function and dysfunction of α-synuclein: mouse lines for modulation of endogenous Snca gene expression. *Sci. Rep.* **5**, 16615; doi: 10.1038/srep16615 (2015).

## Supplementary Material

Supplementary Information
