# Supplementary material for: A novel resource for studying function and dysfunction of α-synuclein: mouse lines for modulation of endogenous Snca gene expression
Source: Sci Rep. 2015 Nov 13;5:16615. doi: 10.1038/srep16615 (PMC4643252; doi:10.1038/srep16615)
Supplement: Supplementary Information [file srep16615-s1.pdf]

Supplementary Information

A novel resource for studying function and dysfunction of α-synuclein: mouse lines for modulation of endogenous Snca gene expression

Natalia Ninkina[1,2], Natalie Connor-Robson[1], Alexey A. Ustiugov[1,2], Tatiana V. Tarasova[1,2], Tatyana A. Shelkovnikova[1,2], Vladimir Buchman[1,2,*]

[1]School of Biosciences, Cardiff University, Museum Avenue, Cardiff, CF10 3AX, United Kingdom

[2]Institute of Physiologically Active Compounds Russian Academy of Sciences, 1 Severniy proezd, Chernogolovka 142432, Moscow Region, Russian Federation

Supplementary Figures

Figure S1. Sequences of Snca loci

Mouse Snca locus (exons I – III)

TATGGGTCACTGTGAAAATGCATTATTGGCAGTAATAAAAGAAGAAAAGAAACTAATAAACTAGTGATTTATGCAA
CAGCATAGGTGAACTAACACATCATGCTGACTGGTATAAACAAAGGCCATATACTCCATGGATATGTACAGAATCA
AATAGAATTATAAACATAGTTCAAAGGGATGAAACATTTCCTTTTATCTTTTGAGATTTCACTCAGGTCAGATAAC
TGGCCAGACTGTGTGACTGAAGATAATAGAAACCAGACAGTGCTGATGTTAGGAGCAACACCCTGACCAGTACCGC
TTAGTTTTGCATGCAATGAGTGTTCTAGATATTGAAATAGTCTCTCTTTAAAATGGTATGCTATCACTTGGACTTT
TTCAAAATCTGCAGACACAAAATCAGAGCAGTTCACTCTATAAACTATAATTCAATGTAGAATATCATTTGATGCC
ATCCTGGGTATTTCAGTCATTCTCACATTTATTAATGTGTGCTAGAATGTTCCCAGATGGAAAAACATGAAAAGCT
TAAATCTCTAGAAGGAGAGAAGTCGATAGTGACAGAGTAGCCATGCTGAAGGCACAGAATGATGCTTGTGGAAGCT
GGTGATATTTATGTAGGAATCTTAGTCTCACAACTGTAAATATGTTTAAATGTTTTACATTCTAAAATTTTAGAGG
AGAGGTGTCATCTCAATTCACTTTCTCTTCTATAATAGAAAAAAAAAAAACCTGGCTAAATAGAACATAACTTGGT
AAAGTTCTGAGAGGCAGAAAACCAACGCCCAGACGCAACCAAAACAGGCCTGGCAAAACATTATCCCGAGGAAACG
TTTGTGTCCTCTCATCTGGCTTTAGACTATTGACAAATAGACCCCAAGAAATTGGAAGTCCTCCAGGAATTTGCTG
AGGGAAGGAAAAGGCTGAAGCCTTGTGTCAATTACAGGGTGAGCATGTCTCCCAGGAAGAAATATCAGATATCAGA
TACTTAGTCAGACCTCCTTGCAGAAGAGACTGGAGCGGAGACAGAGACAGTAGCTGGAAGCACACTTTGACCTACT
GCTTAGTCATACATACATCCTGACCTCTATCTAAACAAGATGAACTTGGGGCACTAAACCTCTGTTCCTCTTCTTA
ACGTGGCCACATTGAATTACTCCCATTTCTAGTATTTCACTATTTATATGTCACTTTACCTGGCTGGTTGAGGACA
GGTGTCCTAACTTGGCAGGATGGGGATGCTAGAGCCCAGGATCTAACCCTATCTACTGCAGAGGTGCCACCTTTTC
CTTTAATTTCAAGTAAACATGGTATGTGCCACTAGTGTGTAGGAAGGTTGATTTTTAAAGGGAATAAGAATTGAAG
GCGTTGCTTAAACAGTTAATTTCTGTCACATTACTTGTACTCTGCATTTGTGGTTTTATCTGCCTCCTTCCTTTAT
AGCATGCCAAACAAGCTGCTTGTCCCTTGTTTCAAATGCTTTTTTAGACTTCAATTTATTTATTTATTTATTTATT
TATTTATTTATTTTTCAGGATTCAGAAGTCAACTGACTTCAAGGATCAGAGAAAGCATTCCCTCCTACGACCCCCC
CCCCCTTTTAATACAGTAAACGCTTGATTTAGCTTCCAGTG***CCCAACACAAGTTCAGAATACAAGAAAGGAAAAGC***
AAGGCACTCTGCTGGGGGAGGAGCTTGGCACTCAAATCCACTCTGCTATAAAACAGTGGTATTCTGCTCATCTCAG
AGAGAAGTGGGAACGTGTTAAGTAACACAGAAATTGTCTCAAAGCCTGTGCATCTATCTGCGCGTGTGCTTGGATT
GGAAGAAGAGTCTGTTCGCTGGAGCTCCACGCAGCCAGAAGTCGGAAAGGTAAGAGGTGTGCAAAATCTGCCATTA
AGTAGGGACTAAGGAAGAAACTGCCTGTGATGGTCCCAGAGGGTGAATCCCACAGCCGCTACCTTCCTATCCTGTA
ACTCTATAGTAAGCCACTTTCTCAAGTGCAAAAAAGCCTTGAGGCAGCTGGTTTTCGACGGTTGGGGGATATTTAT
TCCTTGCTCCACAGATGGGGAAAAAAAAATCAGCGTCTGGCAGCCGCTGATTGGTGGAAAAGAAAATGGTGATAGT
GGAGTGGGAATGAGGATTTGCTGAGCCTCCCCCTGCTTCTTCGACCTGTAACTCTTCCTTAGTCGGCTCCCCTTTG
CACCCAGAACCCTTTTAGACTCCTCCGGGGTAAAAACAAATGGAAATCTTAAGCTGTGTGAACAAAAGCAACCCCA
AGGGTGTGTGCTCCCTCTCCATTGCCTGGCTCCGCACACAGACCATTTCAGGCGGTCCAGCTCTCTGGTGTGGCAT
CTGGGCTCGTCCTGGAGGAGGGGGTCGCCTAGAGGAACTGGGAACAGACTGAGGCAGGGAAGGAGGGGGGTGGGGC
AGGAGAGGCGCCAGCTCAAGTTCAGCCACGATAAAACTGAGGGCCCTCTGAACTCGAGGGGAGGCTCAGGCCGTCC
TCTCTTCCTTCCATCCGGGGGAATGTGCTCCAGATACCCACAGCCCTCACGCACCGCACCTCCAACCAACCCGTCC
CCTCCCTAGGAAGAGGAGCGAAGGCACGAGGCAGGCGAGGGGCGGGGAGAGGCGCTGACAAATCAGCTGCGGGGGC
GACGTGAAGGAGCCAGGGAGCCAGAGCGCCCGGCAGCAGGCAGCAGACGGCAGGAGACCAGCAGGTGTTCCCCCTG
CCCCTGCCTGCCCTTGCCTCTTTCATTGAAATTAGATTGGGGAAAACAGGAAGAATCGGAGTTCTTCAGAAGCCTA
GGGAGCCGGTAAGTACCTGTAGATGGGGCAGCTCTGGGGATCTTAGCTAGCCGGAGCAAAGAGCCGGGACGCCTAG
AGAAGACCAACTACAGCTGCTTTGGCGGTGGGGACTGGGCCAGTGCGTGGAAAGTACATCACTCGGCTTTCCTTTC
GCTGGAGACATGCCCTTCCATCCTGTCAAAGCCCGAGGGAAAGGCCAGGTTGCCTGTGGCATCTGCTTTTTCAAGC
GGAAACGCTAGGGTGTTTCATGTTGAGTGCTGGATGGTGGAAGCTTAGTGCTGGGCATTGGGTGGAATTTGAGCAT
CCAACTTTCATGCTCCAACCCCAGGCATTTCAGCTTCTTTCTGTAGAGGAAGAAGGGTGCCTTTGGCCCATGATTA
ATAGAAGTGCAGAGGACAGTAGGCAACAGGTGATAAAGGGTTAATGAGCATGGGGTGCAGGGTCTTCTAGAGGATT
CCAGCTGAGGACAGAGCTTCTTGGTTGGGTGGTGCTCAAGTGAGACTGCTCAAGTGTATGGACAGCGCCTGCTCTG
GGCAGATAGCAGGCAAAGAGCTAGTGGTGGGCAGAAGGTCTTGCAAGATTAGAAAGGCTGGGCTTCAAGCAGTTCC
CTACTTCTAGATTAAACAGTTCCCCTCCCTTCCTTCTCCAAAGACTGACTCCTCTCTGGGTCTTTTATCCTCTTGC
CCCCACTCCATCTCTGTACGCCCACCTCCCATGTTCCTTTTCTAGATAGTCTTTTTACTTTGAATGTAACCTTTGG
GCCCTGGGAACTTGATGGGGTAGAGGATGCCCACCTCCCCTTCTGCAACTCTTCTTCTGAAATATGTATGTAAGAG
CAGTCGAATGATCAAACTAGATCCATCCCATCCTTAAGTGACATGACTTTTTCCTAGTATTGAGTGACATAACTCA
ACAATCAATCAACACTGTGCCCAGCACCCCCACATCCCCCCACCCAAGAAATCACACTTACACCAGGACTTGGGGG
AAGGCATACTGATTTTTCCCCCTCAATTTCCTTTCTTTCTCTAGCTGTTTTAAACCTTATTATTATTATTTTTTTA
CCCAAATTTTCTAATTCAAAATGTATTCTGTATTCTCTAGTGTGGAGCAAAAATACATCTTTAGCC***ATG***GATGTGT
TCATGAAAGGACTTTCAAAGGCCAAGGAGGGAGTTGTGGCTGCTGCTGAGAAAACCAAGCAGGGTGTGGCAGAGGC
AGCTGGAAAGACAAAAGAGGGAGTCCTCTATGTAGGTAGGTAGTGACACTGTGACTAATGAATTGGGGTGGCTGGT
GTGTGGTGTCTGATTCGTGTGCATCACAGCTTCTCAGAAGAGTGACAGCTGTGTGGAGGTGAGAGAATATGAACCT
GCATATTAGCTCTCAGAAACAAACAGGGACAATGTTTTCTGTCCTTAGATTCATTAATCTTGTTATTTATGTAGGT
TTTTTATTTGGTTTTCTGTTTCTGTGTATGAATACACTGAATTTTAAAAATTGGCAACCCATGAAAAATAACCAAG
AATATGCTTATGAATCAAAGACATGTATGGCAGTAAGCCTGGTGGCATTTGGGAAGTGGAGGCCCAAGGACCAGGA
GTTGATGGTCATCTTCAGCTACACAGAGAATTTGATGCCAGCCTGAACTATGTGAGAACACACACACACACACACA
CACACACACACACACTCACACTCTCTCTCTCTCTCTCTCTCTCTCTCTCTCTCTCACACACACACACACTCA

CACACACACACAATACACACACACACACTCTCTCTTACACACACACATACACACATACACACATACACACACACAC
ATACACACACACACACACTCACACACACACACAAAGAAATAAAGAAATAAAGGAAGGAAGGAAGGAAGGAAGAAAG
AAAGAAAGAAAGAGAAAGAAAGAAAGAAAGAAAGAAAGAAAGAAAGAAAGAAAGAAAGAAAGAAA***GTGAGCCACAAGTAC***
TCATGGGACTTTGATTTCTTTCATCATCACTATAGGTAATACCTGCTAAGTTTAATAAATTATAAAGCTTTAAACA
ATAGTTTTGCATAATTTTATTTTACAACTGTGAAAATACAACTCCTTTGACCCTCAAATAGAAGAAAGAAAGCAAG
TCTTCTTTGGTGGATCTCCTTTTAGGGATCACTTGGTCAGTGGGAACAGCGGGACTTAAGGAACTTCAGAAATGTT
TGTTTAGTTCACCTGTCAGAGATCATACATGCTGAACAGTAAGAGGTTGATATTTAGTGCCATTTTCTGCCTGACT
GTACACATTGAAAGGAAGGCCAACACTCCCTTTCTCTGTCTTTCCCTGTGTTAAATTGGCTGTAACTTTACAAATC
CCTTCTAGTACTTTCATGGAAGGAATAGACACCCATGCACACATGCTTATCCCCAGCAGAGACACAGGTGCACATG
GGAGCACAGTTGCAGGGTTCATCTACCTCTCTTTCCTCCTGTGAACACTGTTTCCACCTTCTTAGGAGGGCATCTC
TCTTGGTGGAAGACTCAGGGTAAACATTCAGGCTGAAAAGGAGCAGAACAGGTGGCAAAAGTGATGCAGATGCTAC
CCAGAGTACCAATCGGGGGAAGCCATGCTGACCCTCCAAACGATCAGTGAGGAATTGATACTTGTAAACATTTTCA
TGAATGTGTCTTTTCATTGAAGTTTCTAGCAGATCACCTTTCCTAATTCTTCACAGAATAATTTTACATTGAATTA
ATTCTCTTTTTCTACTTAAAACATCCTTTCAGAAAGTCTTGTAATGAGTATTGTAAGAGAAGGGTGTCAATGAGCT
AATTTTAGAGTGTTTTTTTTTTTAATGAATTGTGAAGTATAATGTTTTAGATAGAATTCAGAATATAAAAGCAGTAA
TTTGTAGATTTGGGGAAAAACTCAATTCTTCCACAACTACAGGCTTGTGACTGATTTTTTTTTTTTTTACTTCAGT
TGCTTAAGAAACATATCTGTAGATCACTAATTTAAAGCAAATTTAGAAGTTGTTGAATATTAATTTAGTATATTAC
TCTTTCTGGATAATAAATGGATTTTGTCAAGCAGAACACTTCTTTGTTTTTATTGTTAATTTTGAGTTTGGGCAAA
TAAAGTGATTATATTTTTCAAAGATTAATTTTGTTGGTCTCTGTGAGGCCATTATATTGAAAGTGTAATTTTAATA
TGTCTAATATTATTAAAATTATCAATGTCTGTTATTATATTTAAAACATGTTTAATTAATCAATTGCTTATTATGT
TCTGGAATCTAATTAAAAGCTGAACACATGCATAGAGTTTGGGATGAAGAGTAATGTGTGAAGATAAGAATGATAG
CTCAGATATTTGTCAACTTCTGTTAATGTTCCAACACATATTAGAAAATCTGTCATAGATAATCAGCTGTACTGTT
GGCTATACTGATTATTGCTTAGATAATCAACTGTGCTGTTAAAGTATGAAAACAACCATAGGCAAAAAACAGTGTG
ACTCTGCCTCTGTCTTTATTGACTCAGAGACTATAGAGAAATGAAAGGAATGTAGACTCTGGACTTGACTTGATAC
AGACAGAAATTTAATTCAAGCCACATGATTTCTGCCTTTAGCATCTGCAGGAGGTAACTTGATATCTTTGAGTCTC
CTCCCCTTTTTCACATACACATAGTTCATAAAAATGCAACTGCTTTGTAAAGTTACTAAAGTTATGTAGTTAAGGT
AGTAACTGAGTGCACTTTCATATTTAGGAAACTTGAATCTTGTCAGAGAAGTTGTTCAATCTATCTGTTACTCAGT
CAACCTAATTTCTTACTTTTTATCCAAGATATGAAACTATTATTAATACCTAACCTGAAGGATTAGAAATAATCTG
GACTTTGGACATAGCTCCCGTGGCACAGTGCTTGTCTGCCAGCATGCAGCCCTGGGTTCTATTCCCGTACCAGAAA
AACAAAAGATTAAAAATAAAAGGTTAGAAGTAATCAAAGAAAAACAATGTAAACTTCAGCACTTATGGCTGAAAAG
GCTTGGCAGAAGTCTCATCTCATCTCTAATAACAAATGCCTTGGACAACTGCCTTTCAATGAATTGAAGACCTGCC
ATACTAATCAGTGTGCTGATTGTCTCTGTGATATTTGCACAAAAAATTCAATTAACATATTTTAGCTTCATAATCA
ACAGTCTCAATGGCGTGATGTATAATTATAAATTGAATTTAAAGTCAAAAAGTTTTCTTCACTTCATGTTAGTTTT
ATTAATACTATAAAGAAAATCACCTTCAAGTTCTGTTTCACTGCCTGGTGAAGAGCTGTGGTCACACATCTAACTC
CTAAGTCTCACATGTGAGACTTAACTACATGTTGCTAAGTAGTCAGCATATAAACCAATGATATGACTCATTTCTC
ACATTCCTCTTAGGTCCGTATCCTTGTAATATTCCAAATAAACAAGACAGGGTGGGGTGGAAGGCAGGGTACATTT
CTAGGCTCAGAGAAGCCATTATTATATTGTTCCCCAGCTTCCATATCTTACTTCTTATTTGCTACTTGATGACTAA
TTTTTTTTTGCTATATCTTATCAGTTAGATCTCACCTGTAAACTGAAGATAAACTATCATTTATAACTTAGCTGAT
AATTAGGATAACAAAGGTGAGAGGTATGGTTTGAGATACAGGGCCTTCAAGACTCATTTGTCTTTCATTAAAGAGG
CATTCCATGATTTTACCAAACGTCAAATTCTCTGTTACTGCTGAGGCAAAGAAGACAGACAAGAGACCAGCCAGTG
AGCATTAGTTTTCCTTGGTCATGCTTTTTTTTTAATTGGGTATTTTATGTATTTACATTTTAAACGTTATCCCCTA
TTCTATTCTAAACCCCTTCCCTGGCTTCTATGAGAATGCTCCCCTGCCACCCATATACTTTCACCTCACGGCCCTG
GCATTCCCCTACACTAGCGAATCCAGCCTTCACAGGTCCAAGGGCTCTTCTTCTATTGATGCCAGACAATGCCATC
CTCTACTACATATGCAGCTGGAGCTATGGGTTCCTCTATGTGTACTTTTTGGTTGGTGGTTTATGGGAGCTCTGGA
GGGTCTTGTTGATTGATATTCCTATGGGGTTTCAAAATGGTTGGCTTCCAGCATCCGAATCTGTATTGATCAGGCT
CTAGCCGAGCCTCTCAGGAGACAGCTGTATCAGGCTCCTTTCAGCAAGCAGTTCTTGGTATTAGCAGTAGTGTCTG
GGTTTGGTGTCTGCAAATAAAATGAAGCCTTTCCTTCAGTCTCTGCTCCACTCTTTGTCCCTGTGTCTCCTCTAGA
CAGGAGCTCTTAAAGCTTGTTGTAGTGAAGATGATACAGAAGAGTTGAGTTCTCTCACGCAAGCTGTTCTACTACT
TGTGCAGGGTGCCCTGCCCACCACCATTTCCAGTTGTGATGTGAATAGCACCTGTCTCATAAAGCACAACTTAAAC
ACCTGTGATTGCAGTGCATAAATTAATAGTAATTATTCGAGGTACAAACTTTACTGCTAGCACTTCACCCTAAAAA
TTATCGCAAAAATAATGAAAGCCCAATGTAATTGGTGACTACATTAAACTACTTCTTTCAGAATTTGTCCATGAGC
TGCCACTTTCCATCTGTTACAAGATTTGCACAAAAAGCAGCACCTGTGGGTGTGCTGTCTTTTGTAACCTGCTAAT
AAATCCGTGTGATATTTTTACAGACACACATCTCAGAAAGGGGAAACTGACCAGCTGAGGTGAAGTCACATCAAGG
CAATAAAGTGCAAAATCCTGGGAGCAATTTGTTTATAGAAAAATAACAGCTGAATATTCAGATTGCAGAAATGTAA
ATTGAATATTTAATAATTTTGGAAATAGCAATTGGTTCATACCCGGGTTAGTGTATATCAACTTGAAAGAAAGTAG
AGCTAGCATATGTGGTCTCTAGTGTAGTCCTAGATAGTATGTACACACTTCAGGGTCAGGAGGTAAATGTACAAGC
TTACACTGAGGATTGTGACATATCAGAAGCCATTGTCTCAGAGGAAGTAATGCCTTCTTAACCCCATGCTAAAAGA
ACTATCAGAGTCAGATCGCGGCATGAAGAGTTGTGGTGGTTTGAATAGGAATGCCACCCAGAGTCTCATGAACCTG
GTACCAGCCAGTGGTACTGTTTGGGAAGGAATATGCAGTGTAGCCTTGGTAGCCGAGGTATGTCACAGGGAGAGGC
AGTGAAGGTTTAATAGCCACCCATCATTCCCAGTGTACTCTTGGTCCCCTGCTTTTGGATCAATATGCAAGCTCTC
CATTGTTCCTGCTGCCCTTCCCTTCCTACTCCACTGTGGATTCTAACACACCCAATGTTTTAGGACATGAAAAAGA
TACCCACACCGTAAAGGCATATGCAATGAGAAGAAGGCAAGCTTTGTTGAAACTACTTAATAAGCACATTGTTTTT
GCAAAAATTAAAAATTCTAAACTACAAAATATAAAATAAATATTAGCTTTAACATTTTATCATTTCCCAACATACT
TGTGTTTAATAATTTGACTCATAGCCCCCTCACCATCCACTGCTTATACAGTTTCCCCATTCATTGTTAGGTTCTG
TACACTGATCAGCTCAGCTTGTCCTCACAGCTCTACAGTCCCTTGCAAAATGAGCAGTGCCTATGAAATGCATGCA
GACAGCACCCATGCAGAACACATATCCGTTCCTGCTAACAAGTGTGCCTTTCTCTCTGCGCTGCTTCTAGTGCGGT
GATCTTTCCTGTGCTTTCAGCTTCAGCTTCTCCTTCAGAGGCATTTGTATGGGTAAGAACAAGAGTTTGCACCATG
TCTGTATCATGCATTCAACAGTACTGAGGGCTTTACTTCAACGATTTCCTTTTATTCTTTTGCCAAGATCATGATG
CAGATTTCGTTAACCTTTAGTGAAGTGAAGAGTTAAATCTGGACTCTGTATCGGGGTGGGGGTGGGTGGTTCTTTA
TTTTCAAAATAAAAGTTCCTACATATGCTTTTTTAATTAATGAGGGTTTAATTGACTCCTTTCTAAAATATTATTT

TAAATAAAATAGACAAAAATTCTCTTAAGGCTATATGTATATATCTTCAAAACTATTTACTAAATAATTTAACATA
CTTTTGTACATGTACTTAGGTTATCTTATTGATCATATTATTCAGCTTGTAGAAATGCACATCTGAATTTTAAGCA
ATTTTGGAATTAGAAATTACCTCATAGTTAGTGTTTGTCAACTTGACAGGAAGTAGAGATATGTGGGAAGAGGACA
TAACATTTGAGGAAATGTCTACCTCTGATTTACCCATAGTAATGTTTGTGAGGATATTTTCCTGATTGACAACTGA
TGGAGGAGCACCCAGCCCACTGTGGGTGGCACCACCCCTAGGCAGGTATTTTTGAGTGTTATAAGAAAGCAGGCTG
AGCAAGATATGGAGAGCAAACCAGTGAGCAGCATTTTCCCGAGGTCTCCACATCAGAGCCTGCCTCCAGGTTCCTG
CCATGCTTGGAGTTTCTACTTTTGGTTCCCTCGATAATGAACTTCCAAACTGGAAGCTGAGAAATCTCCTTTTCCA
CACTTTGTGTTTGGTCACAGTGTTCATCACCAAACAGAAGACTTTGATTGGCAAGTTAGTTATGTACAGGGAATGT
TTACTCTAAATGTTGGTATCTGTACTTTATGACTGAGCAGTTGGCTTCTAGGAAGCTATGTATATGATATAGTTTT
TGTACTAGTTTTTTTTCCTCTTCTTGTTTTCTGTCCATGTAGCAAGACATTTTTTTTCTTCTCAAATAGTGCATTT
TTAAAATCCACTATTTTAAAGTTTTAAAATTCCCCCCCCCACATGCTGGCCTAAGTCTTTTTCAGCTTATATGTC
CTCATGTCCTTTTTATCCTTTGCATTCTTCTGTGTCTAGATAAGATTATTTTAGTTAATGTTCCTCTCTCCATCTC
TTTAGTCCTTTCTTCCTTGGTTTCTTGGTAATATTGGGGATCAAATTTAGGTCCTTAAACATCAGAAAACAGTGCT
GCACTAAGAACTATGTCTTTATCCCTATAGGATAGCTTTCACTTAAAAATGTGTATTTTTATATGTATGTATATAT
AATATGCATGTATATTGTATATATATACAGATATATAAAAATTTTATGCATGCAGATAAAATTATCAGTATTGATT
GTACAAAGTGAGAGGCCTCATTATGATGTGTGGGTCTCCCCTTCCTTGGAGGTAATTGGCAACTGGCCTAATAGGC
TGAGGGGAGCAGAGGCGGTTCAGGCTTCAGACTACCATAAGTATGATGGATTGACTTCTGGGATCAGCTTTAGTGA
GACATAACAACTTAGACAGTGCTAGGGATTTCTGGGTGGGTGTAGATTATTGGCTAGGTTCGAGGTGCTGAGGATG
TGTCATTTAAAGAAAGAGGAATTCCAGGAATTATTGGGAGAGAGGTTGTTGAATCTGTAATCTGGCCATTGACAAC
ATGATTGTCTTTATAGGTGAGGGACATAGAGGCCTGATGCCACAGCAAGTAGACTAAGAATAGGGAGAGAGTGATC
CTAACTCCTGCCTGTCTAAGGATGAGATTTGTCAGCATCTTGATCCCGTCTCACTCTTGCTCCAGGCTAGCTCTGC
TGGCTGCACATTCTCACAATGATCTTCCCACAGATGCATTTAATATACAAGGTTATAGCCACCCTTCTATTACTAG
TTTTTTATTATTATTTGTAGAGATAATGCTTTTTATATTTTTATTTGCTTTGTTATTCCTGCGCTTTCATTTTTGT
TGTGTATACTCATTGTTCATGGTTCCATTCCATAAGGACATTTTTATATAAGTATATAGAACACGATTTTTCACAA
TTCATGAATGTATTTTGATCATAACTCCTCTCCTTTATTCTTTCTCCCCCTTGCTCTTCCTCTCCACTTCTTTAGT
AAAGCCCAGCTGCTTTTGCGTACTTTTTATCACTCTATGCATATCTGGGAGAAAAAATGATGCTATGTTTTTCTCT
GTGAGCTGGGTCATTTCATTGAACATGATGATCTGACTTTTTCCCTACACATATCATAATTTCCTTCTTTTTTATT
TCCGACTACAAGTCAATTATGAAACCCAGTGTGTGGAGAATTCTTAAAAAGTAAGAAATAAAATTTCCAGCCATGC
CACTTCTGTGCAACCACCAGAGCCACCATACAAGAATGATGTACTGCATACCATGCATATTTGACTATTCAACCAT
AGAGTGTTATGGAAGCAACCCAGATACTCACCAGTGGATGACTGGAAGAAGAGACTCTGGTATAAATCAAAACCAG
AGTTTTTCAAATGAACCTTAAATCTCCAAACTATTTAATCAAATGGTGGTCATTATACTGAAATTTTAAGCATTAG
AAAGATTATTTTTAAAATGATTAACAAACTTACTTTTAATAATATGTGCAATAGCTATTTCTTTGTTTAGTAATGG
CTCAAGGCATAGGTGAAATTCTTATCTTACATACAGTCCTAGTTTGAAAGTAACATGCTGTTACTTAATAATTATG
CAAATCACTTAATTATGATTTTTAGTTTCCTTATGTATGAAATGGGTATTGAATGGCTGCATCAGAGATGATGTGA
GGTCAATCTGTACCAGGGGTTGGGCAGACGCTGATATCTTCTTTCCTCTCCCTTTTTTGTTGTGGATTGTGCAGTC
TCTGCTCTGTTGTGCTTTTACAGCATTCTCAGGTCTGCACAGAGAATCTTACTATGCCTGTGTTATCTTCCCTTTC
CTTCTCTCTGTAAATTGATGAAGAAAGCATCAAGCAAGGGTTATGTAAAGAGTCGTTATGTTTTGTGCATTGTGTT
TTATGTTTTATCTGATAAATAAAGGCACAAAACTTTTACCAGTGTTGCCTCTGGTGCAGTTCCCATCCATGTTCAC
ATTGTGTGGTCAAGCTACACATATCTGTTGCCTCTAACATATGTCAGATCTTTATGATATTAACCACTGAAGCTTG
TAGCCTTTTGAGATCCACAGTGCCCAGTTGCTGTCTATTATCTCCCAGGTGGAACAGCACAGGAGCTTCATACTGC
TGACTAACTCAACTGGCTACCCACTAAACCCTCTCCAGGCTTCCCTCCTGAACTCAACCTGGATAGGCTGGTGGTA
GCTTTCCTCTGGGGTGGTGGCCAGATCCCCCCCACTTTAGTGATTTCTGAGTGTGATTGGTGGTTGTTAGTCTTCT
GAAGTTATCTTTGTACATTCCCTTCTGAATATTGAGAATTTTTAATTGGCTGCTGTAAATTGAAGGACAGTTTAAT
ATTTATGCGTTCAATTTCTTTGTTCTTTAGGTTCCAAAACTAAGGAAGGAGTGGTTCATGGAGTGACAACAGGTAA
GCTCTGTTGTCTTTTATCCAGGGGTGATATGCCGAATGCCTTCTAGGCTAAATTAACTTGATGCTTATACTTCAAG

Blue highlighted – exons I - III of the Snca gene (the start codon in exon II is in ***ITALIC BOLD***)

ITALIC BOLD – arms used in the targeted construct to achieve homologous recombination

Yellow highlighted – Southern hybridisation probe A, see Figure 1.

Green highlighted – Southern hybridisation probe B, see Figure 1.

LIGHT GRAY HIGHLIGHTED – repetitive region deleted in the targeting construct to increase the rate of homologous recombination.

Snca flox(neo) locus

GTAGGAAGGTTGATTTTTAAAGGGAATAAGAATTGAAGGCGTTGCTTAAACAGTTAATTTCTGTCACATTACTTGT
ACTCTGCATTTGTGGTTTTATCTGCCTCCTTCCTTTATAGCATGCCAAACAAGCTGCTTGTCCCTTGTTTCAAATG
CTTTTTTAGACTTCAATTTATTTATTTATTTATTTATTTATTTATTTATTTTTCAGGATTCAGAAGTCAACTGACT
TCAAGGATCAGAGAAAGCATTCCCTCCTACGACCCCCCCCCCCTTTTAATACAGTAAACGCTTGATTTAGCTTCCA
GTG***CCCAACACAAGTTCAGAATACAAGAAAGGAAAAGCAAGGCACTCTGCTGGGGGAGGAGCTTGGCACTCAAATC***

CACTCTGCTATAAAACAGTGGTATTCTGCTCATCTCAGAGAGAAGTGGGAACGTGTTAAGTAACACAGAAATTGTC
TCAAAGCCTGTGCATCTATCTGCGCGTGTGCTTGGATTGGAAGAAGAGTCTGTTCGCTGGAGCTCCACGCAGCCAG
AAGTCGGAAAGGTAAGAGGTGTGCAAAATCTGCCATTAAGTAGGGACTAAGGAAGAAACTGCCTGTGATGGTCCCA
GAGGGTGAATCCCACAGCCGCTACCTTCCTATCCTGTAACTCTATAGTAAGCCACTTTCTCAAGTGCAAAAAAGCC
TTGAGGCAGCTGGTTTTCGACGGTTGGGGGATATTTATTCCTTGCTCCACAGATGGGGAAAAAAAAATCAGCGTCT
GGCAGCCGCTGATTGGTGGAAAAGAAAATGGTGATAGTGGAGTGGGAATGAGGATTTGCTGAGCCTCCCCCTGCTT
CTTCGACCTGTAACTCTTCCTTAGTCGGCTCCCCTTTGCACCCAGAACCCTTTTAGACTCCTCCGGGGTAAAAACA
AATGGAAATCTTAAGCTGTGTGAACAAAAGCAACCCCAAGGGTGTGTGCTCCCTCTCCATTGCCTGGCTCCGCACA
CAGACCATTTCAGGCGGTCCAGCTCTCTGGTGTGGCATCTGGGCTCGTCCTGGAGGAGGGGGTCGCCTAGAGGAAC
TGGGAACAGACTGAGGCAGGGAAGGAGGGGGGTGGGGCAGGAGAGGCGCCAGCTCAAGTTCAGCCACGATAAAACT
GAGGGCCCTCTGAACTCGAGGGGAGGCTCAGGCCGTCCTCTCTTCCTTCCATCCGGGGGAATGTGCTCCAGATACC
CACAGCCCTCACGCACCGCACCTCCAACCAACCCGTCCCCTCCCTAGGAAGAGGAGCGAAGGCACGAGGCAGGCGA
GGGGCGGGGAGAGGCGCTGACAAATCAGCTGCGGGGGCGACGTGAAGGAGCCAGGGAGCCAGAGCGCCCGGCAGCA
GGCAGCAGACGGCAGGAGACCAGCAGGTGTTCCCCCTGCCCCTGCCTGCCCTTGCCTCTTTCATTGAAATTAGATT
GGGGAAAACAGGAAGAATCGGAGTTCTTCAGAAGCCTAGGGAGCCGGTAAGTACCTGTAGATGGGGCAGCTCTGGG
GATCTTAGCTAGCCGGAGCAAAGAGCCGGGACGCCTAGAGAAGACCAACTACAGCTGCTTTGGCGGTGGGGACTGG
GCCAGTGCGTGGAAAGTACATCACTCGGCTTTCCTTTCGCTGGAGACATGCCCTTCCATCCTGTCAAAGCCCGAGG
GAAAGGCCAGGTTGCCTGTGGCATCTGCTTTTTCAAGCGGAAACGCTAGGGTGTTTCATGTTGAGTGCTGGATGGT
GGAAGCTTAGTGCTGGGCATTGGGTGGAATTTGAGCATCCAACTTTCATGCTCCAACCCCAGGCATTTCAGCTTCT
TTCTGTAGAGGAAGAAGGGTGCCTTTGGCCCATGATTAATAGAAGTGCAGAGGACAGTAGGCAACAGGTGATAAAG
GGTTAATGAGCATGGGGTGCAGGGTCTTCTAGAGGATTCCAGCTGAGGACAGAGCTTCTTGGTTGGGTGGTGCTCA
AGTGAGACTGCTCAAGTGTATGGACAGCGCCTGCTCTGGGCAGATAGCAGGCAAAGAGCTAGTGGTGGGCAGAAGG
TCTTGCAAGATTAGAAAGGCTGGGCTTCAAGCAGTTCCCTACTTCTAGATTAAACAGTTCCCCTCCCTTCCTTCTC
CAAAGACTGACTCCTCTCTGGGTCTTTTATCCTCTTGCCCCCACTCCATCTCTGTACGCCCACCTCCCATGTTCCT
TTTCTAGATAGTCTTTTTACTTTGAATGTAACCTTTGGGCCCTGGGAACTTGATGGGGTAGAGGATGCCCACCTCC
GGATCCataacttcgtatagcatacattatacgaagttatccatggTCTAGCCCTTCTGCAACTCTTCTTCTGAAA
TATGTATGTAAGAGCAGTCGAATGATCAAACTAGATCCATCCCATCCTTAAGTGACATGACTTTTTCCTAGTATTG
AGTGACATAACTCAACAATCAATCAACACTGTGCCCAGCACCCCCACATCCCCCCACCCAAGAAATCACACTTACA
CCAGGACTTGGGGGAAGGCATACTGATTTTTCCCCCTCAATTTCCTTTCTTTCTCTAGCTGTTTTAAACCTTATTA
TTATTATTTTTTTACCCAAATTTTCTAATTCAAAATGTATTCTGTATTCTCTAGTGTGGAGCAAAAATACATCTTT
AGCC***ATG***GATGTGTTCATGAAAGGACTTTCAAAGGCCAAGGAGGGAGTTGTGGCTGCTGCTGAGAAAACCAAGCAG
GGTGTGGCAGAGGCAGCTGGAAAGACAAAAGAGGGAGTCCTCTATGTAGGTAGGTAGTGACACTGTGACTAATGAA
TTGGGGTGGCTGGTGTGTGGTGTCTGATTCGTGTGCATCACAGCTTCTCAGAAGAGTGACAGCTGTGTGGAGGTGA
GAGAATATGAACCTGCATATTAGCTCTCAGAAACAAACAGGGACAATGTTTTCTGTCCTTAGATTCATTAATCTTG
TTATTTATGTAGGTTTTTTATTTGGTTTTCTGTTTCTGTGTATGAATACACTGAATTTTAAAAATTGGCAACCCAT
GAAAAATAACCAAGAATATGCTTATGAATCAAAGACATGTATGGCAGTAAGCCTGgaagttcctattctctagaaa
gtataggaacttcGCTAGAGTCAGCTTCTGATGGAATTAGAACTTGGCAAAACAATACTGAGAATGAAGTGTATGT
GGAACAGAGGCTGCTGATCTCGTTCTTCAGGCTATGAAACTGACACATTTGGAAACCACAGTACTTAGAACCACAA
AGTGGGAATCAAGAGAAAAACAATGATCCCACGAGAGATCTATAGATCTATAGATCATGAGTGGGAGGAATGAGCT
GGCCCTTAATTTGGTTTTGCTTGTTTAAATTATGATATCCAACTATGAAACATTATCATAAAGCAATAGTAAAGAG
CCTTCAGTAAAGAGCAGGCATTTATCTAATCCCACCCCACCCCCACCCCCGTAGCTCCAATCCTTCCATTCAAAAT
GTAGGTACTCTGTTCTCACCCTTCTTAACAAAGTATGACAGGAAAAACTTCCATTTTAGTGGACATCTTTATTGTT
TAATAGATCATCAATTTCTGCAGACTTACAGGACGGATCGATCCCCTCAGAAGAACTCGTCAAGAAGGCGATAGAA
GGCGATGCGCTGCGAATCGGGAGCGGCGATACCGTAAAGCACGAGGAAGCGGTCAGCCCATTCGCCGCCAAGCTCT
TCAGCAATATCACGGGTAGCCAACGCTATGTCCTGATAGCGGTCCGCCACACCCAGCCGGCCACAGTCGATGAATC
CAGAAAAGCGGCCATTTTCCACCATGATATTCGGCAAGCAGGCATCGCCATGGGTCACGACGAGATCATCGCCGTC
GGGCATGCGCGCCTTGAGCCTGGCGAACAGTTCGGCTGGCGCGAGCCCCTGATGCTCTTCGTCCAGATCATCCTGA
TCGACAAGACCGGCTTCCATCCGAGTACGTGCTCGCTCGATGCGATGTTTCGCTTGGTGGTCGAATGGGCAGGTAG
CCGGATCAAGCGTATGCAGCCGCCGCATTGCATCAGCCATGATGGATACTTTCTCGGCAGGAGCAAGGTGAGATGA
CAGGAGATCCTGCCCCGGCACTTCGCCCAATAGCAGCCAGTCCCTTCCCGCTTCAGTGACAACGTCGAGCACAGCT
GCGCAAGGAACGCCCGTCGTGGCCAGCCACGATAGCCGCGCTGCCTCGTCCTGCAGTTCATTCAGGGCACCGGACA
GGTCGGTCTTGACAAAAAGAACCGGGCGCCCCTGCGCTGACAGCCGGAACACGGCGGCATCAGAGCAGCCGATTGT
CTGTTGTGCCCAGTCATAGCCGAATAGCCTCTCCACCCAAGCGGCCGGAGAACCTGCGTGCAATCCATCTTGTTCA
ATGGCCGATCCCATATTGGCTGCAGGTCGAAAGGCCCGGAGATGAGGAAGAGGAGAACAGCGCGGCAGACGTGCGC
TTTTGAAGCGTGCGAGAATGCCGGGCCTCGGGAGGACCTTCGCGCCCGCCCCGCCCCTGAGCCCGCCCCTGAGCCC
GCCCCCGGACCCACCCCTTCCCAGCCTCTGAGCCCAGAAAGCGAAGGAGCAAAGCTGCTATTGGCCGCTGCCCCAA
AGGCCTACCCGCTTCCATTGCTCAGCGGTGCTGTCCATCTGCACGAGACTAGTGAGACGTGCTACTTCCATTTGTC
ACGTCCTGCACGACGCGAGCTGCGGGGCGGGGGGAACTTCCTGACTAGGGGAGGAGTAGAAGGTGGCGCGAAGGG
GCCACCAAAGAACGGAGCCGGTTGGCGCTACCGGTGGATGTGGAATGTGTGCGAGGCCAGAGGCCACTTGTGTAGC
GCCAAGTGCCAGCGGGGCTGCTAAAGCGCATGCTCCAGACTGCCTTGGGAAAAGCGCCTCCCCTACCCGGTAGAAT
TGACCTGCAGG**gaagttcctattctctagaaagtataggaacttcCCAAataacttcgtatagcatacattatacg**
aagttatGTCGAC***GTGAGCCACAAGTACTCATGGGACTTTGATTTCTTTCATCATCACTATAGGTAATACCTGCTA***
AGTTTAATAAATTATAAAGCTTTAAACAATAGTTTTGCATAATTTTATTTTACAACTGTGAAAATACAACTCCTTT
GACCCTCAAATAGAAGAAAGAAAGCAAGTCTTCTTTGGTGGATCTCCTTTTAGGGATCACTTGGTCAGTGGGAACA
GCGGGACTTAAGGAACTTCAGAAATGTTTGTTTAGTTCACCTGTCAGAGATCATACATGCTGAACAGTAAGAGGTT
GATATTTAGTGCCATTTTCTGCCTGACTGTACACATTGAAAGGAAGGCCAACACTCCCTTTCTCTGTCTTTCCCTG
TGTTAAATTGGCTGTAACTTTACAAATCCCTTCTAGTACTTTCATGGAAGGAATAGACACCCATGCACACATGCTT
ATCCCCAGCAGAGACACAGGTGCACATGGGAGCACAGTTGCAGGGTTCATCTACCTCTCTTTCCTCCTGTGAACAC
TGTTTCCACCTTCTTAGGAGGGCATCTCTCTTGGTGGAAGACTCAGGGTAAACATTCAGGCTGAAAAGGAGCAGAA
CAGGTGGCAAAAGTGATGCAGATGCTACCCAGAGTACCAATCGGGGGAAGCCATGCTGACCCTCCAAACGATCAGT

GAGGAATTGATACTTGTAAACATTTTCATGAATGTGTCTTTTCATTGAAGTTTCTAGCAGATCACCTTTCCTAATT
CTTCACAGAATAATTTTACATTGAATTAATTCTCTTTTTCTACTTAAAACATCCTTTCAGAAAGTCTTGTAATGAG
TATTGTAAGAGAAGGGTGTCAATGAGCTAATTTTAGAGTGTTTTTTTTTTAATGAATTGTGAAGTATAATGTTTTA
GATAGAATTCAGAATATAAAAGCAGTAATTTGTAGATTTGGGAAAAACTCAATTCTTCCACAACTACAGGCTTGT
GACTGATTTTTTTTTTTTTTACTTCAGTTGCTTAAGAAACATATCTGTAGATCACTAATTTAAAGCAAATTTAGAA
GTTGTTGAATATTAATTTAGTATATTACTCTTTCTGGATAATAAATGGATTTTGTCAAGCAGAACACTTCTTTGTT
TTTATTGTTAATTTTGAGTTTGGGCAAATAAAGTGATTATATTTTTCAAAGATTAATTTTGTTGGTCTCTGTGAGG
CCATTATATTGAAAGTGTAATTTTAATATGTCTAATATTATTAAAATTATCAATGTCTGTTATTATATTTAAAACA
TGTTTAATTAATCAATTGCTTATTATGTTCTGGAATCTAATTAAAAGCTGAACACATGCATAGAGTTTGGGATGAA
GAGTAATGTGTGAAGATAAGAATGATAGCTCAGATATTTGTCAACTTCTGTTAATGTTCCAACACATATTAGAAAA
TCTGTCATAGATAATCAGCTGTACTGTTGGCTATACTGATTATTGCTTAGATAATCAACTGTGCTGTTAAAGTATG
AAAACAACCATAGGCAAAAAACAGTGTGACTCTGCCTCTGTCTTTATTGACTCAGAGACTATAGAGAAATGAAAGG
AATGTAGACTCTGGACTTGACTTGATACAGACAGAAATTTAATTCAAGCCACATGATTTCTGCCTTTAGCATCTGC
AGGAGGTAACTTGATATCTTTGAGTCTCCTCCCCTTTTTCACATACACATAGTTCATAAAAATGCAACTGCTTTGT
AAAGTTACTAAAGTTATGTAGTTAAGGTAGTAACTGAGTGCACTTTCATATTTAGGAAACTTGAATCTTGTCAGAG
AAGTTGTTCAATCTAT

red lowercase – loxP sites

underlined lowercase – FRT sites

ITALIC BOLD – arms used in the targeted construct to achieve homologous recombination

Dark yellow highlighted – neo-cassette

Blue highlighted – exons I and II of the Snca gene (the start codon in exon II is in ***ITALIC BOLD***)

Snca floxΔneo locus

GTAGGAAGGTTGATTTTTAAAGGGAATAAGAATTGAAGGCGTTGCTTAAACAGTTAATTTCTGTCACATTACTTGT
ACTCTGCATTTGTGGTTTTATCTGCCTCCTTCCTTTATAGCATGCCAAACAAGCTGCTTGTCCCTTGTTTCAAATG
CTTTTTTAGACTTCAATTTATTTATTTATTTATTTATTTATTTATTTTTCAGGATTCAGAAGTCAACTGACT
TCAAGGATCAGAGAAAGCATTCCCTCCTACGACCCCCCCCCCCTTTTAATACAGTAAACGCTTGATTTAGCTTCCA
GTG***CCCAACACAAGTTCAGAATACAAGAAAGGAAAAGCAAGGCACTCTGCTGGGGGAGGAGCTTGGCACTCAAATC***
CACTCTGCTATAAAACAGTGGTATTCTGCTCATCTCAGAGAGAAGTGGGAACGTGTTAAGTAACACAGAAATTGTC
TCAAAGCCTGTGCATCTATCTGCGCGTGTGCTTGGATTGGAAGAAGAGTCTGTTCGCTGGAGCTCCACGCAGCCAG
AAGTCGGAAAGGTAAGAGGTGTGCAAAATCTGCCATTAAGTAGGGACTAAGGAAGAAACTGCCTGTGATGGTCCCA
GAGGGTGAATCCCACAGCCGCTACCTTCCTATCCTGTAACTCTATAGTAAGCCACTTTCTCAAGTGCAAAAAAGCC
TTGAGGCAGCTGGTTTTCGACGGTTGGGGGATATTTATTCCTTGCTCCACAGATGGGGAAAAAAAAATCAGCGTCT
GGCAGCCGCTGATTGGTGGAAAAGAAAATGGTGATAGTGGAGTGGGAATGAGGATTTGCTGAGCCTCCCCCTGCTT
CTTCGACCTGTAACTCTTCCTTAGTCGGCTCCCCTTTGCACCCAGAACCCTTTTAGACTCCTCCGGGGTAAAAACA
AATGGAAATCTTAAGCTGTGTGAACAAAAGCAACCCCAAGGGTGTGTGCTCCCTCTCCATTGCCTGGCTCCGCACA
CAGACCATTTCAGGCGGTCCAGCTCTCTGGTGTGGCATCTGGGCTCGTCCTGGAGGAGGGGGTCGCCTAGAGGAAC
TGGGAACAGACTGAGGCAGGGAAGGAGGGGGGTGGGGCAGGAGAGGCGCCAGCTCAAGTTCAGCCACGATAAAACT
GAGGGCCCTCTGAACTCGAGGGGAGGCTCAGGCCGTCCTCTCTTCCTTCCATCCGGGGGAATGTGCTCCAGATACC
CACAGCCCTCACGCACCGCACCTCCAACCAACCCGTCCCCTCCCTAGGAAGAGGAGCGAAGGCACGAGGCAGGCGA
GGGGCGGGGAGAGGCGCTGACAAATCAGCTGCGGGGGCGACGTGAAGGAGCCAGGGAGCCAGAGCGCCCGGCAGCA
GGCAGCAGACGGCAGGAGACCAGCAGGTGTTCCCCCTGCCCCTGCCTGCCCTTGCCTCTTTCATTGAAATTAGATT
GGGGAAAACAGGAAGAATCGGAGTTCTTCAGAAGCCTAGGGAGCCGGTAAGTACCTGTAGATGGGGCAGCTCTGGG
GATCTTAGCTAGCCGGAGCAAAGAGCCGGGACGCCTAGAGAAGACCAACTACAGCTGCTTTGGCGGTGGGGACTGG
GCCAGTGCGTGGAAAGTACATCACTCGGCTTTCCTTTCGCTGGAGACATGCCCTTCCATCCTGTCAAAGCCCGAGG
GAAAGGCCAGGTTGCCTGTGGCATCTGCTTTTTCAAGCGGAAACGCTAGGGTGTTTCATGTTGAGTGCTGGATGGT
GGAAGCTTAGTGCTGGGCATTGGGTGGAATTTGAGCATCCAACTTTCATGCTCCAACCCCAGGCATTTCAGCTTCT
TTCTGTAGAGGAAGAAGGGTGCCTTTGGCCCATGATTAATAGAAGTGCAGAGGACAGTAGGCAACAGGTGATAAAG
GGTTAATGAGCATGGGGTGCAGGGTCTTCTAGAGGATTCCAGCTGAGGACAGAGCTTCTTGGTTGGGTGGTGCTCA
AGTGAGACTGCTCAAGTGTATGGACAGCGCCTGCTCTGGGCAGATAGCAGGCAAAGAGCTAGTGGTGGGCAGAAGG
TCTTGCAAGATTAGAAAGGCTGGGCTTCAAGCAGTTCCCTACTTCTAGATTAAACAGTTCCCCTCCCTTCCTTCTC
CAAAGACTGACTCCTCTCTGGGTCTTTTATCCTCTTGCCCCCACTCCATCTCTGTACGCCCACCTCCCATGTTCCT
TTTCTAGATAGTCTTTTTACTTTGAATGTAACCTTTGGGCCCTGGGAACTTGATGGGGTAGAGGATGCCCACCTC**C**
GGATCCataacttcgtatagcatacattatacgaagttatccatggTCTAGCCCTTCTGCAACTCTTCTTCTGAAA
TATGTATGTAAGAGCAGTCGAATGATCAAACTAGATCCATCCCATCCTTAAGTGACATGACTTTTTCCTAGTATTG
AGTGACATAACTCAACAATCAATCAACACTGTGCCCAGCACCCCCACATCCCCCCACCCAAGAAATCACACTTACA
CCAGGACTTGGGGGAAGGCATACTGATTTTTCCCCCTCAATTTCCTTTCTTTCTCTAGCTGTTTTAAACCTTATTA
TTATTATTTTTTTACCCAAATTTTCTAATTCAAAATGTATTCTGTATTCTCTAGTGTGGAGCAAAAATACATCTTT

AGCC***ATG***GATGTGTTCATGAAAGGACTTTCAAAGGCCAAGGAGGGAGTTGTGGCTGCTGCTGAGAAAACCAAGCAG
GGTGTGGCAGAGGCAGCTGGAAAGACAAAAGAGGGAGTCCTCTATGTAGGTAGGTAGTGACACTGTGACTAATGAA
TTGGGGTGGCTGGTGTGTGGTGTCTGATTCGTGTGCATCACAGCTTCTCAGAAGAGTGACAGCTGTGTGGAGGTGA
GAGAATATGAACCTGCATATTAGCTCTCAGAAACAAACAGGGACAATGTTTTCTGTCCTTAGATTCATTAATCTTG
TTATTTATGTAGGTTTTTTATTTGGTTTTCTGTTTCTGTGTATGAATACACTGAATTTTAAAAATTGGCAACCCAT
GAAAAATAACCAAGAATATGCTTATGAATCAAAGACATGTATGGCAGTAAGCCTG**gaagttcctattctctagaaa**
gtataggaacttcCCAAataacttcgtatagcatacattatacgaagttatGTCGAC***GTGAGCCACAAGTACTCAT***
GGGACTTTGATTTCTTTCATCATCACTATAGGTAATACCTGCTAAGTTTAATAAATTATAAAGCTTTAAACAATAG
TTTTGCATAATTTTATTTTACAACTGTGAAAATACAACTCCTTTGACCCTCAAATAGAAGAAAGAAAGCAAGTCTT
CTTTGGTGGATCTCCTTTTAGGGATCACTTGGTCAGTGGGAACAGCGGGACTTAAGGAACTTCAGAAATGTTTGTT
TAGTTCACCTGTCAGAGATCATACATGCTGAACAGTAAGAGGTTGATATTTAGTGCCATTTTCTGCCTGACTGTAC
ACATTGAAAGGAAGGCCAACACTCCCTTTCTCTGTCTTTCCCTGTGTTAAATTGGCTGTAACTTTACAAATCCCTT
CTAGTACTTTCATGGAAGGAATAGACACCCATGCACACATGCTTATCCCCAGCAGAGACACAGGTGCACATGGGAG
CACAGTTGCAGGGTTCATCTACCTCTCTTTCCTCCTGTGAACACTGTTTCCACCTTCTTAGGAGGGCATCTCTCTT
GGTGGAAGACTCAGGGTAAACATTCAGGCTGAAAAGGAGCAGAACAGGTGGCAAAAGTGATGCAGATGCTACCCAG
AGTACCAATCGGGGGAAGCCATGCTGACCCTCCAAACGATCAGTGAGGAATTGATACTTGTAAACATTTTCATGAA
TGTGTCTTTTCATTGAAGTTTCTAGCAGATCACCTTTCCTAATTCTTCACAGAATAATTTTACATTGAATTAATTC
TCTTTTTCTACTTAAAACATCCTTTCAGAAAGTCTTGTAATGAGTATTGTAAGAGAAGGGTGTCAATGAGCTAATT
TTAGAGTGTTTTTTTTTTAATGAATTGTGAAGTATAATGTTTTAGATAGAATTCAGAATATAAAAGCAGTAATTTG
TAGATTTGGGGAAAAACTCAATTCTTCCACAACTACAGGCTTGTGACTGATTTTTTTTTTTTTTTACTTCAGTTGCT
TAAGAAACATATCTGTAGATCACTAATTTAAAGCAAATTTAGAAGTTGTTGAATATTAATTTAGTATATTACTCTT
TCTGGATAATAAATGGATTTTGTCAAGCAGAACACTTCTTTGTTTTTATTGTTAATTTTGAGTTTGGGCAAATAAA
GTGATTATATTTTTCAAAGATTAATTTTGTTGGTCTCTGTGAGGCCATTATATTGAAAGTGTAATTTTAATATGTC
TAATATTATTAAAATTATCAATGTCTGTTATTATATTTAAAACATGTTTAATTAATCAATTGCTTATTATGTTCTG
GAATCTAATTAAAAGCTGAACACATGCATAGAGTTTGGGATGAAGAGTAATGTGTGAAGATAAGAATGATAGCTCA
GATATTTGTCAACTTCTGTTAATGTTCCAACACATATTAGAAAATCTGTCATAGATAATCAGCTGTACTGTTGGCT
ATACTGATTATTGCTTAGATAATCAACTGTGCTGTTAAAGTATGAAAACAACCATAGGCAAAAAACAGTGTGACTC
TGCCTCTGTCTTTATTGACTCAGAGACTATAGAGAAATGAAAGGAATGTAGACTCTGGACTTGACTTGATACAGAC
AGAAATTTAATTCAAGCCACATGATTTCTGCCTTTAGCATCTGCAGGAGGTAACTTGATATCTTTGAGTCTCCTCC
CCTTTTTCACATACACATAGTTCATAAAAATGCAACTGCTTTGTAAAGTTACTAAAGTTATGTAGTTAAGGTAGTA
ACTGAGTGCACTTTCATATTTAGGAAACTTGAATCTTGTCAGAGAAGTTGTTCAATCTAT

red lowercase – loxP sites

underlined lowercase – FRT site

ITALIC BOLD – arms used in the targeted construct to achieve homologous recombination

Blue highlighted – exons I and II of the Snca gene (the start codon in exon II is in ***ITALIC BOLD***)

Snca Δflox locus

GTAGGAAGGTTGATTTTTAAAGGGAATAAGAATTGAAGGCGTTGCTTAAACAGTTAATTTCTGTCACATTACTTGT
ACTCTGCATTTGTGGTTTTATCTGCCTCCTTCCTTTATAGCATGCCAAACAAGCTGCTTGTCCCTTGTTTCAAATG
CTTTTTTAGACTTCAATTTATTTATTTATTTATTTATTTATTTATTTATTTTTCAGGATTCAGAAGTCAACTGACT
TCAAGGATCAGAGAAAGCATTCCCTCCTACGACCCCCCCCCCCTTTTAATACAGTAAACGCTTGATTTAGCTTCCA
GTG***CCCAACACAAGTTCAGAATACAAGAAAGGAAAAGCAAGGCACTCTGCTGGGGGAGGAGCTTGGCACTCAAATC***
CACTCTGCTATAAAACAGTGGTATTCTGCTCATCTCAGAGAGAAGTGGGAACGTGTTAAGTAACACAGAAATTGTC
TCAAAGCCTGTGCATCTATCTGCGCGTGTGCTTGGATTGGAAGAAGAGTCTGTTCGCTGGAGCTCCACGCAGCCAG
AAGTCGGAAAGGTAAGAGGTGTGCAAAATCTGCCATTAAGTAGGGACTAAGGAAGAAACTGCCTGTGATGGTCCCA
GAGGGTGAATCCCACAGCCGCTACCTTCCTATCCTGTAACTCTATAGTAAGCCACTTTCTCAAGTGCAAAAAAGCC
TTGAGGCAGCTGGTTTTCGACGGTTGGGGGATATTTATTCCTTGCTCCACAGATGGGAAAAAAAAATCAGCGTCT
GGCAGCCGCTGATTGGTGGAAAAGAAAATGGTGATAGTGGAGTGGGAATGAGGATTTGCTGAGCCTCCCCCTGCTT
CTTCGACCTGTAACTCTTCCTTAGTCGGCTCCCCTTTGCACCCAGAACCCTTTTAGACTCCTCCGGGGTAAAAACA
AATGGAAATCTTAAGCTGTGTGAACAAAAGCAACCCCAAGGGTGTGTGCTCCCTCTCCATTGCCTGGCTCCGCACA
CAGACCATTTCAGGCGGTCCAGCTCTCTGGTGTGGCATCTGGGCTCGTCCTGGAGGAGGGGGTCGCCTAGAGGAAC
TGGGAACAGACTGAGGCAGGGAAGGAGGGGGGTGGGGCAGGAGAGGCGCCAGCTCAAGTTCAGCCACGATAAAACT
GAGGGCCCTCTGAACTCGAGGGGAGGCTCAGGCCGTCCTCTCTTCCTTCCATCCGGGGGAATGTGCTCCAGATACC
CACAGCCCTCACGCACCGCACCTCCAACCAACCCGTCCCCTCCCTAGGAAGAGGAGCGAAGGCACGAGGCAGGCGA
GGGGCGGGGAGAGGCGCTGACAAATCAGCTGCGGGGGCGACGTGAAGGAGCCAGGGAGCCAGAGCGCCCGGCAGCA
GGCAGCAGACGGCAGGAGACCAGCAGGTGTTCCCCCTGCCCCTGCCTGCCCTTGCCTCTTTCATTGAAATTAGATT
GGGGAAAACAGGAAGAATCGGAGTTCTTCAGAAGCCTAGGGAGCCGGTAAGTACCTGTAGATGGGGCAGCTCTGGG
GATCTTAGCTAGCCGGAGCAAAGAGCCGGGACGCCTAGAGAAGACCAACTACAGCTGCTTTGGCGGTGGGGACTGG
GCCAGTGCGTGGAAAGTACATCACTCGGCTTTCCTTTCGCTGGAGACATGCCCTTCCATCCTGTCAAAGCCCGAGG

GAAAGGCCAGGTTGCCTGTGGCATCTGCTTTTTCAAGCGGAAACGCTAGGGTGTTTCATGTTGAGTGCTGGATGGT
GGAAGCTTAGTGCTGGGCATTGGGTGGAATTTGAGCATCCAACTTTCATGCTCCAACCCCAGGCATTTCAGCTTCT
TTCTGTAGAGGAAGAAGGGTGCCTTTGGCCCATGATTAATAGAAGTGCAGAGGACAGTAGGCAACAGGTGATAAAG
GGTTAATGAGCATGGGGTGCAGGGTCTTCTAGAGGATTCCAGCTGAGGACAGAGCTTCTTGGTTGGGTGGTGCTCA
AGTGAGACTGCTCAAGTGTATGGACAGCGCCTGCTCTGGGCAGATAGCAGGCAAAGAGCTAGTGGTGGGCAGAAGG
TCTTGCAAGATTAGAAAGGCTGGGCTTCAAGCAGTTCCCTACTTCTAGATTAAACAGTTCCCCTCCCTTCCTTCTC
CAAAGACTGACTCCTCTCTGGGTCTTTTATCCTCTTGCCCCCACTCCATCTCTGTACGCCCACCTCCCATGTTCCT
TTTCTAGATAGTCTTTTTACTTTGAATGTAACCTTTGGGCCCTGGGAACTTGATGGGGTAGAGGATGCCCACCTCC
GGATCCataacttcgtatagcatacattatacgaagttatGTCGAC***GTGAGCCACAAGTACTCATGGGACTTTGAT***
TTCTTTCATCATCACTATAGGTAATACCTGCTAAGTTTAATAAATTATAAAGCTTTAAACAATAGTTTTGCATAAT
TTTATTTTACAACTGTGAAAATACAACTCCTTTGACCCTCAAATAGAAGAAAGAAAGCAAGTCTTCTTTGGTGGAT
CTCCTTTTAGGGATCACTTGGTCAGTGGGAACAGCGGGACTTAAGGAACTTCAGAAATGTTTGTTTAGTTCACCTG
TCAGAGATCATACATGCTGAACAGTAAGAGGTTGATATTTAGTGCCATTTTCTGCCTGACTGTACACATTGAAAGG
AAGGCCAACACTCCCTTTCTCTGTCTTTCCCTGTGTTAAATTGGCTGTAACTTTACAAATCCCTTCTAGTACTTTC
ATGGAAGGAATAGACACCCATGCACACATGCTTATCCCCAGCAGAGACACAGGTGCACATGGGAGCACAGTTGCAG
GGTTCATCTACCTCTCTTTCCTCCTGTGAACACTGTTTCCACCTTCTTAGGAGGGCATCTCTCTTGGTGGAAGACT
CAGGGTAAACATTCAGGCTGAAAAGGAGCAGAACAGGTGGCAAAAGTGATGCAGATGCTACCCAGAGTACCAATCG
GGGGAAGCCATGCTGACCCTCCAAACGATCAGTGAGGAATTGATACTTGTAAACATTTTCATGAATGTGTCTTTTC
ATTGAAGTTTCTAGCAGATCACCTTTCCTAATTCTTCACAGAATAATTTTACATTGAATTAATTCTCTTTTTCTAC
TTAAAACATCCTTTCAGAAAGTCTTGTAATGAGTATTGTAAGAGAAGGGTGTCAATGAGCTAATTTTAGAGTGTTT
TTTTTTTAATGAATTGTGAAGTATAATGTTTTAGATAGAATTCAGAATATAAAAGCAGTAATTTGTAGATTTGGGG
AAAAACTCAATTCTTCCACAACTACAGGCTTGTGACTGATTTTTTTTTTTTTTACTTCAGTTGCTTAAGAAACATA
TCTGTAGATCACTAATTTAAAGCAAATTTAGAAGTTGTTGAATATTAATTTAGTATATTACTCTTTCTGGATAATA
AATGGATTTTGTCAAGCAGAACACTTCTTTGTTTTTATTGTTAATTTTGAGTTTGGGCAAATAAAGTGATTATATT
TTTCAAAGATTAATTTTGTTGGTCTCTGTGAGGCCATTATATTGAAAGTGTAATTTTAATATGTCTAATATTATTA
AAATTATCAATGTCTGTTATTATATTTAAAACATGTTTAATTAATCAATTGCTTATTATGTTCTGGAATCTAATTA
AAAGCTGAACACATGCATAGAGTTTGGGATGAAGAGTAATGTGTGAAGATAAGAATGATAGCTCAGATATTTGTCA
ACTTCTGTTAATGTTCCAACACATATTAGAAAATCTGTCATAGATAATCAGCTGTACTGTTGGCTATACTGATTAT
TGCTTAGATAATCAACTGTGCTGTTAAAGTATGAAAACAACCATAGGCAAAAAACAGTGTGACTCTGCCTCTGTCT
TTATTGACTCAGAGACTATAGAGAAATGAAAGGAATGTAGACTCTGGACTTGACTTGATACAGACAGAAATTTAAT
TCAAGCCACATGATTTCTGCCTTTAGCATCTGCAGGAGGTAACTTGATATCTTTGAGTCTCCTCCCCTTTTTCACA
TACACATAGTTCATAAAAATGCAACTGCTTTGTAAAGTTACTAAAGTTATGTAGTTAAGGTAGTAACTGAGTGCAC
TTTCATATTTAGGAAACTTGAATCTTGTCAGAGAAGTTGTTCAATCTAT

red lowercase – loxP site

ITALIC BOLD – arms used in the targeted construct to achieve homologous recombination

Blue highlighted – exon I of the Snca gene

Supplementary Materials and Methods

PCR genotyping protocols

A standard amplification protocol (95°C for 2 min followed by 35 cycles of 95°C for 20 sec; 60°C for 20 sec; 72°C for 30 sec) was used for genotyping of all loci shown below.

<u>NSE-Cre transgene</u>

Primers:

Cre/ER1 5' – ATACCGGAGATCATGCAAGC – 3'

Cre/ER2 5' – CAAAGCCTGGCACTCTCTTT – 3'

Product: 393 bp (no band for wildtype animals)

Actin-Flp transgene

Primers:

ActFlpFor 5' – CACTGATATTGTAAGTAGTTTGC – 3'

ActFlpRev 5' – CTAGTGCGAAGTAGTGATCAGG – 3'

Product: 720 bp (no band for wildtype animals)

Rosa26

Primers:

mRosaFor 5' – ATTGCTTGTGATCCGCCTCGGAGT – 3'

mRosaRev1 5' – AGAGGCATTCATGGGAGTGGAAAG – 3'

mRosaInsRev1 5' – CTTTTACTGGCCTGCTCCCTTATC – 3'

Products: wildtype – 577 bp; Rosa26-stop-lacZ – 450 bp

Snca$^{flox(neo)}$ locus and Snca$^{flox\Delta neo}$ locus (upstream junction)

Primers:

A_Int1For 5' – TGCTGGGCACAGTGTTGATTG – 3'

A_Int1Rev 5' – AAAGGCTGGGCTTCAAGCAG – 3'

Products: wildtype – 354 bp; floxed Snca – 406 bp

Snca$^{flox(neo)}$ locus (downstream junction)

Primers:

alphaE1 5' – GACATGTATGGCAGTAAGCC – 3'

Cre Rev 5' – CATGAGTACTTGTGGCTCAC – 3'

neoB' 5' – CTGAAGAACGAGATCAGCAGCCT – 3'

Products: wildtype – 442 bp; floxed(neo) Snca – 149 bp

Snca$^{flox\Delta neo}$ locus (downstream junction)

Primers:

alphaE1 5' – GACATGTATGGCAGTAAGCC – 3'

alphaLA 5' – CCACTGACCAAGTGATCCCT – 3'

Products: wildtype – 632 bp; floxed-Δneo-Snca – 310 bp

Snca$^{\Delta flox}$ locus

Primers:

A_Int1For 5' – TGCTGGGCACAGTGTTGATTG – 3'

A_Int1Rev 5' – AAAGGCTGGGCTTCAAGCAG – 3'

Cre Rev 5' – CATGAGTACTTGTGGCTCAC – 3'

Products: wildtype – 354 bp; floxed Snca – 406 bp; Δfloxed Snca – 280 bp
